# Special Issue: Microbial Degradation of Xenobiotics

**DOI:** 10.3390/microorganisms8040487

**Published:** 2020-03-30

**Authors:** Yuji Nagata

**Affiliations:** Graduate School of Life Sciences, Tohoku University, Sendai 980-8577, Japan; aynaga@ige.tohoku.ac.jp

Xenobiotics are released into the environment by human activities, and they often cause problems such as environmental pollution, since most such compounds cannot be readily degraded, and have harmful effects on human beings and the natural ecosystem. However, some microorganisms that degrade man-made xenobiotics have been isolated. Most of these aerobic xenobiotics-degrading bacterial strains use xenobiotics as their sole source of carbon and energy, and thus they are excellent models for studying the adaptation and evolution of bacteria in the environment.

Recent genome analyses of bacterial strains that degrade xenobiotics have strongly suggested that they indeed emerged relatively recently by gathering genes for the degradation of xenobiotics, and mobile genetic elements played important roles in the recruitment of the genes [1]. However, the origin of the genes and the evolutionary processes of such bacterial strains remain largely unknown. Ongoing comprehensive genome and metagenome analyses may provide some insights into these mysteries, and the genes for the degradation of xenobiotics can be used as probes to reveal novel mechanisms for the evolution of microorganisms. In addition, enzymes for the degradation of xenobiotics are good materials for studies on protein evolution, since generally they have promiscuous activities, and their properties change dramatically with a small number of mutations [2]. On the other hand, the importance of microbial consortia and symbiosis for the degradation of xenobiotics in the environment has also been suggested [3], and thus studies on xenobiotics degradation may provide some novel concepts in the field of microbial ecology.

This issue gathers 13 articles dealing with various aspects of the microbial degradation of xenobiotics. Four of them deal with the bacterial strains that degrade monocyclic phenolic compounds [4], polylactic acid [5], and naphthalene [6], and those that accumulate perfluorohexane sulfonate [7]. Two are dedicated to bacterial consortia degrading diesel [8] and dioxane [9]. Two focus on the enzymes for degradation of haloalkanes [10] and bisphenols [11]. Three articles are related to “indirect” factors that are necessary or important for the microbial degradation of xenobiotics, i.e., transcriptional regulation [12], transporters that are involved in the transport of xenobiotic compounds across the outer membrane [13], and mobile genetic elements [14]. The last two articles address metabolic engineering [15] and the bioreactors [16] necessary for practical application.
